# Hidden Diversity in Sardines: Genetic and Morphological Evidence for Cryptic Species in the Goldstripe Sardinella, *Sardinella gibbosa* (Bleeker, 1849)

**DOI:** 10.1371/journal.pone.0084719

**Published:** 2014-01-08

**Authors:** Rey C. Thomas, Demian A. Willette, Kent E. Carpenter, Mudjekeewis D. Santos

**Affiliations:** 1 Genetic Fingerprinting Laboratory, National Fisheries Research and Development Institute, Quezon City, Metro Manila, Philippines; 2 Department of Ecology and Evolutionary Biology, University of California Los Angeles, Los Angeles, California, United States of America; 3 Department of Biological Sciences, Old Dominion University, Norfolk, Virginia, United States of America; College of Charleston, United States of America

## Abstract

Cryptic species continue to be uncovered in many fish taxa, posing challenges for fisheries conservation and management. In *Sardinella gibbosa*, previous investigations revealed subtle intra-species variations, resulting in numerous synonyms and a controversial taxonomy for this sardine. Here, we tested for cryptic diversity within *S. gibbosa* using genetic data from two mitochondrial and one nuclear gene regions of 248 individuals of *S. gibbosa*, collected from eight locations across the Philippine archipelago. Deep genetic divergence and subsequent clustering was consistent across both mitochondrial and nuclear markers. Clade distribution is geographically limited: Clade 1 is widely distributed in the central Philippines, while Clade 2 is limited to the northernmost sampling site. In addition, morphometric analyses revealed a unique head shape that characterized each genetic clade. Hence, both genetic and morphological evidence strongly suggests a hidden diversity within this common and commercially-important sardine.

## Introduction

Accurately defining the limits of a species is essential to studying its biology, ecology, conservation, and management [1]–[3]. Species are typically categorized according to gross morphology [4]. However, species diversity can be masked by a lack of obvious morphological differences between cryptic species [5]. Cryptic species are morphologically similar but genetically distinct lineages, and are often overlooked by species identification using gross morphology alone [6]. Genetic differentiation can be used to distinguish morphologically similar lineages [7]–[9], in which the genotypic-clustering species definition is utilized to identify cryptic species [10]–[12]. Cryptic species are widely distributed across different taxa and geographies in the marine realm [13]–[14], occurring either in allopatry [15], [16] or sympatry [8], [17] as sister species [16], or a result of convergent evolution [18]. In the Indo-West Pacific, cryptic species of widely distributed reef fishes contribute significantly to overall marine biodiversity [19]. Thus, inaccurate species delimitation would overlook cryptic species and underestimate biodiversity, a fact that can lead to flawed conservation and management strategies [5], [20].

Marine small pelagic fishes comprise the majority of world's landed fish catch [21], and members of the family Clupeidae contribute significantly to this volume [22]. The Clupeidae reach their highest diversity in the Indo-West Pacific [23], a region also proposed to be the geographic origin of this family [24]. Within the Indo-West Pacific, the goldstripe sardinella *Sardinella gibbosa* (Bleeker, 1849) is among the most abundant and widespread marine pelagic species. It has a distribution that extends from the East African coast to Taiwan, the Philippines, Indonesia and Northern Australia [23]. Historically, it is among the most abundant and commercially important species in the Indo-West Pacific sardine fishery [25], [26]. In particular, the goldstripe sardinella is the second most abundant sardine occurring in the Philippine archipelago [27]. Migratory patterns of *S. gibbosa* have been correlated with the availability and seasonality of planktonic prey in the environment [26], [28], [29]. Biological data from the coast of India suggests a peak spawning period that lasts from early March towards the end of May [26], [30]. However, two distinct length and age groups have been observed [31], and variations in the number of scale striations have been observed in *S. gibbosa* off the South African coast [32]. Morphological classification of this sardine has been complicated by subtle intra-species variations, leading to several recorded synonyms for *S. gibbosa* [23]. Type specimens, now considered synonyms of *S. gibbosa*, were previously known as *Clupea immaculata* (Southern Japan and China) [33], *Fimbriclupea dactyolepis* (Northwest Australia) [34], and *Sardinella taiwanensis* (Taiwan) [35]. Such subtle biological and morphological differences documented in *S. gibbosa* may hint of hidden diversity within the sardine.

The objective of this work has been to explore the possible cryptic species within the goldstripe sardinella in the Philippine archipelago by examining molecular and morphometric data. We investigated the occurrence of cryptic species using genotypic clustering for both mitochondrial and nuclear markers. We also used morphometric variations to test for subtle morphological differentiation between the genetically-partitioned groups. Both genetic and morphological evidence provide strong support for an unexpected multiple-species complex within the common and commercially-important sardine *S. gibbosa* in the Philippine archipelago.

## Materials and Methods

### Sampling

A total of 378 individuals of *S. gibbosa* were collected from sixteen fish markets across the Philippines, Taiwan, Malaysia, Vietnam and Thailand (Table 1). Body coloration, morphometric, and meristic characters were recorded for frozen then thawed samples. Tissue samples and voucher specimens preserved in absolute ethanol were stored at the National Fisheries Research and Development Institute, Quezon City, Philippines.

**Table 1:** Sampling information for *S. gibbosa*.

Location	*N*	Collection date
Sta. Ana, Cagayan (CAG)	50	June, 2012
Atimonan, Quezon (QUE)	30	July, 2011
Balayan Bay, Batangas (BAT)	22	November, 2011
Manila Bay, Manila (MNL)	30	May, 2012
Tacloban, Leyte (LEY)	30	July, 2011
Ilo-Ilo City, Ilo-Ilo (ILO)	30	May, 2011
Banate, Ilo-Ilo (BAN)	32	April, 2013
Puerto Princesa, Palawan (PAL)	24	December, 2012
Kudat, Sabah (KUD)	21	March, 2011
Yilan County, Taiwan (YIL)	1	April, 2011
Nha Trang, Vietnam (NTR)	26	October, 2011
Phu Quouc, Vietnam (PQU)	26	October, 2011
Songkla, Thailand (SON)	4	October, 2011
Surat Thani, Thailand (SUR)	17	October, 2011
Koh Samui, Thailand (KOH)	8	October, 2011
Trang Province, Thailand (TRA)	27	October, 2011
Total	378	

### PCR amplification

Genomic DNA was extracted from muscle tissue samples using either modified Chelex® (Bio-Rad, Hercules, CA) DNA extraction protocol [36], or salting-out method [37]. Approximately 540 bp of the ribosomal 16S gene region are amplified by polymerase chain reaction (PCR) using the primers 16Sar (5′-CGCCTGTTTATCAAAAACAT-3′) and 16Sbr (5′-CCGGTCTGAACTCAGATCACGT-3′) [38]. Additional mitochondrial and nuclear DNA sequences were obtained only for Philippine collections due to mounting sequencing costs and limited time available for this study. The primers CRA (5′-TTCCACCTCTAACTCCCAAAGCTAG-3′) and CRE (5′-CCTGAAGTAGGAACCAGATG-3′) were used to amplify 560 bp of mitochondrial DNA control region [39]. Nuclear DNA was obtained using the primers S7RPEX1F (5′-TGGCCTCTTCCTTGGCCGTC-3′) and S7RPEX2R (5′-AACTCGTCTGGCTTTTCGCC-3′) to amplify 700 bp of the 1st intron of the ribosomal S7 gene [40]. The total volume of the reaction mixture to amplify mtDNA gene regions is 25 µl; consisting of 13.5 µl of nuclease-free water, 2.5 µl of 10x PCR buffer, 2.5 µl of 10 mM dNTP, 2.0 µl of 25 mM MgCl2, 1.0 µl of 5x BSA, 1.25 µl of 10 mM of both primers, and 0.125 µl of *Taq* DNA polymerase. Thermal cycling conditions consisted of an initial denaturation of 94°C for 10 min followed by 38 cycles of DNA denaturing at 94°C for 30 s, primer annealing at 45°C for 45 s, and sequence extension at 72°C for 45 s, ending with a final extension of 72°C for 10 min. For the ribosomal S7 intron, we utilized PCR conditions as previously described [40]. Successful PCR products were purified using ExoSAP-IT® (USB Corp, Cleveland, OH). The reaction mixture consisted of 2 µl of ExoSAP-IT and 22 µl of PCR product, and eventually incubated at 37°C for 15 min followed by another 15 min at 80°C to inactivate the enzyme. Purified PCR products were sent to either Macrogen, Inc. Korea or UC-Berkeley for DNA sequencing. Sequence data was deposited on the public domain database GenBank [Accession numbers pending].

### Phylogenetic reconstruction

Sequences were assembled in Geneious v5.4 [41] and aligned using MUSCLE v3.8.31 [42]. A best-fit nucleotide substitution model was determined using jMODELTEST v2 [43], [44]. Phylogenetic analysis using maximum likelihood (ML) criteria was inferred from MEGA v5.2.1 [45] using the best-fit nucleotide substitution models, namely, Kimura-2-Parameter (K2P) for 16S, three parameter model (TPM) for control region and Hasegawa-Kishino-Yano (HKY) for the S7 intron. Also included in the analysis for outgroup comparison were the closely related taxa, namely, *Sardinella fimbriata, S. hualiensis, S. lemuru, Herkoltsichthys quadrimaculatus* and *Amblygaster sirm* sequences. Further, such species have overlapping geographic distribution with *S. gibbosa* throughout the Indo-West Pacific [23]. However, we excluded *Amblygaster sirm* and *Herklotsichthys quadrimaculatus* as outgroups for control region dataset since they are highly divergent and inclusion of these taxa created large indels in sequence alignment. Allelic state of the nuclear S7 intron was estimated using PHASE v2.1 [46], [47] as implemented in DnaSP v5.0 [48]. The phylogenetic network was inferred using the median-joining network implemented in NETWORK v4.6 [49] using the default settings.

### Morphological analysis

To complement genetic data, variability within *S. gibbosa* from 10 individuals per site was quantified by morphometric measurements representing the head shape. Measurements (in mm) obtained using a Vernier caliper were standard length, snout length (tip of snout to eye), head length (tip of snout to edge of operculum), eye diameter (horizontal diameter), upper jaw length, and post-orbital length (right edge of eye to end of operculum). All measurements were converted into ratios to represent proportion with respect to standard length. A principal component analysis implemented in PC-ORD v4.10 [50] was performed on natural log-transformed ratios which separated morphological variations into linear combinations of variables that describe overall head shape. In addition, analysis of similarities (ANOSIM) and similarity of percentage analysis (SIMPER) were conducted on log-transformed morphometric ratios in Primer v5.2.4 [51] to determine the percentage contribution of morphometric ratios to the overall variations in head shape.

## Results

**Figure 1:**
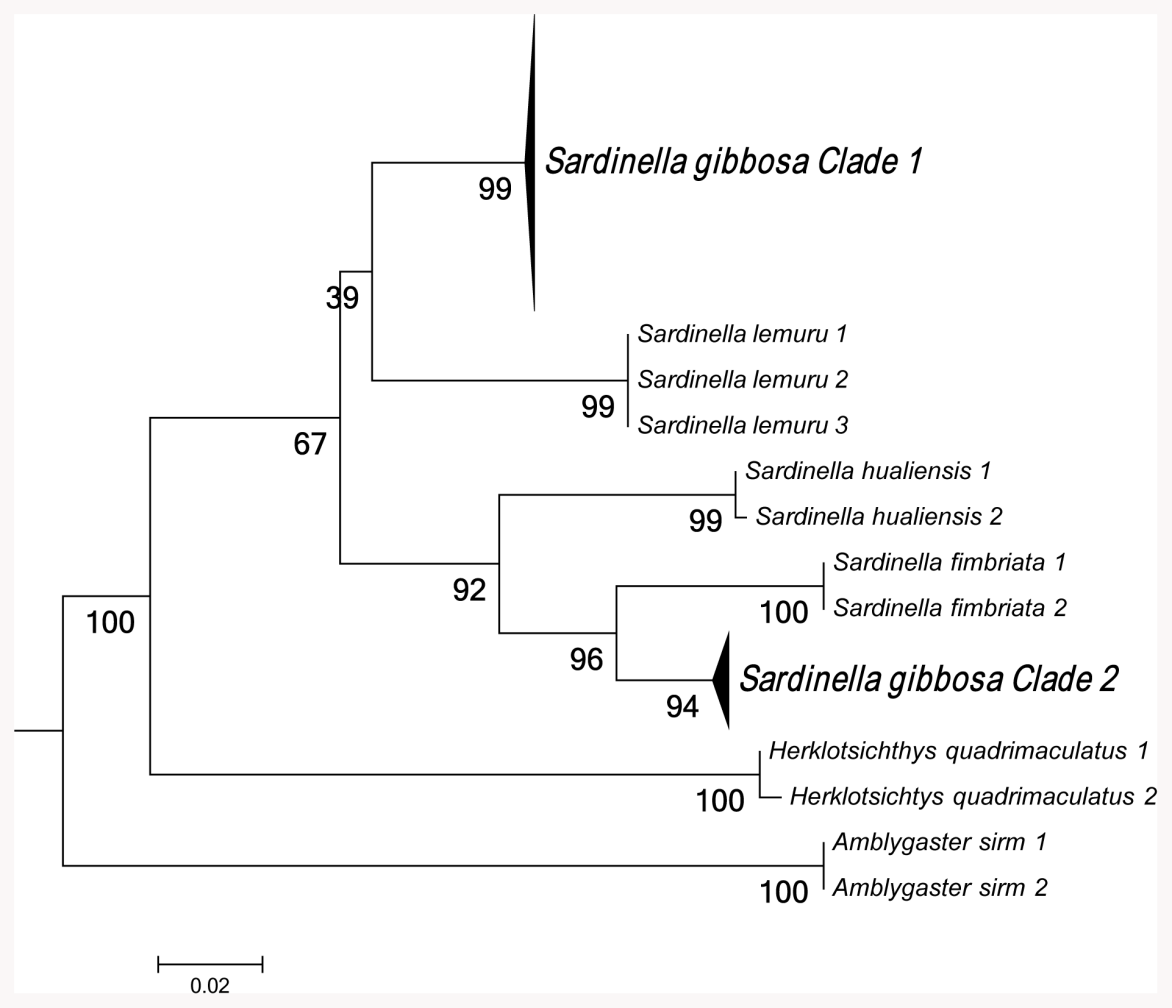
Maximum-likelihood (ML) tree inferred using 16S rRNA sequences. Bootstrap support values were calculated using 1,000 replicates. Sequences of *S. hualiensis* obtained from GenBank were JN580490.1 and JN580479.1.

**Figure 2:**
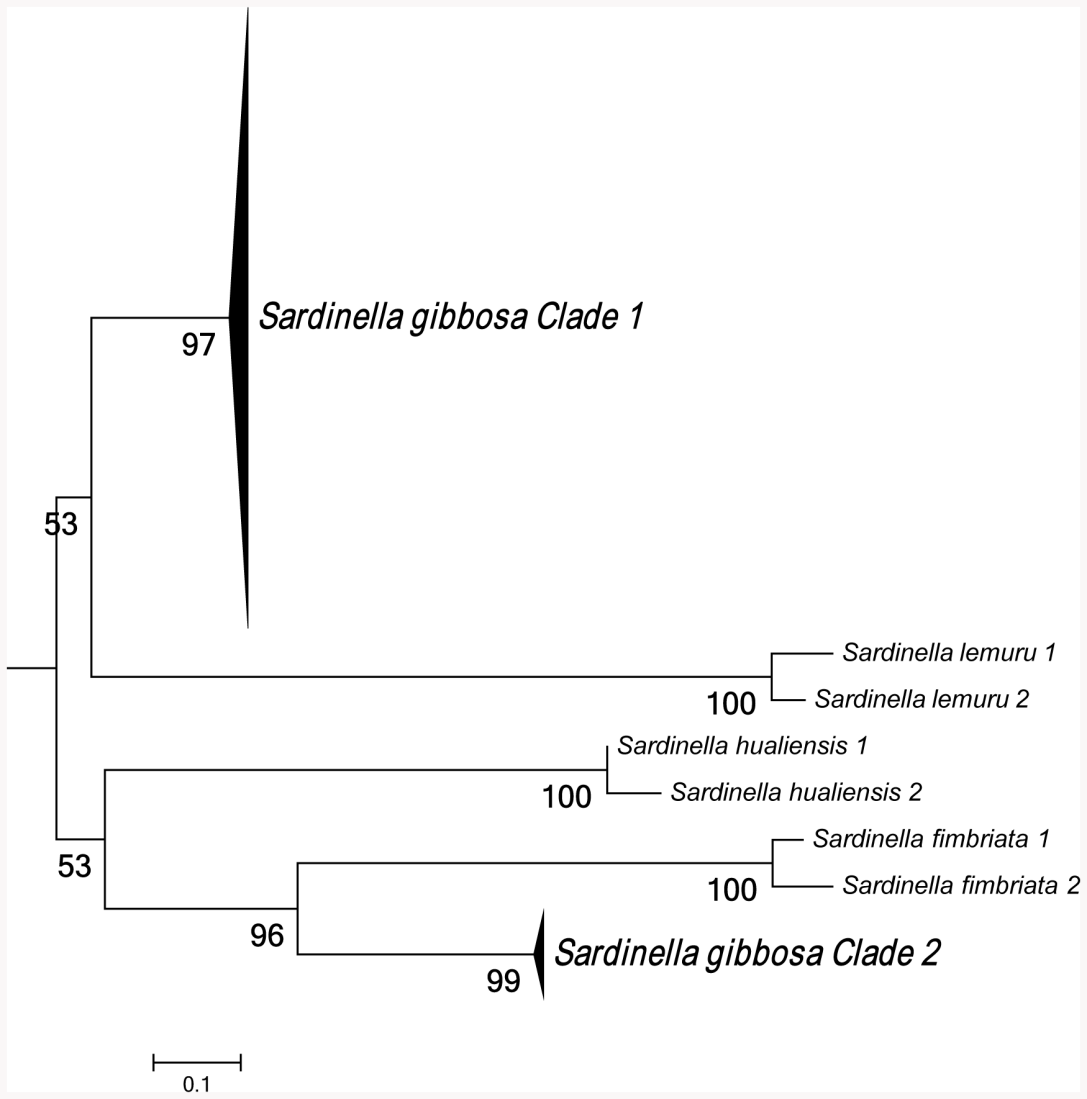
Maximum-likelihood (ML) tree generated from mitochondrial control region sequences. Bootstrap support values were calculated using 1,000 replicates.

**Figure 3:**
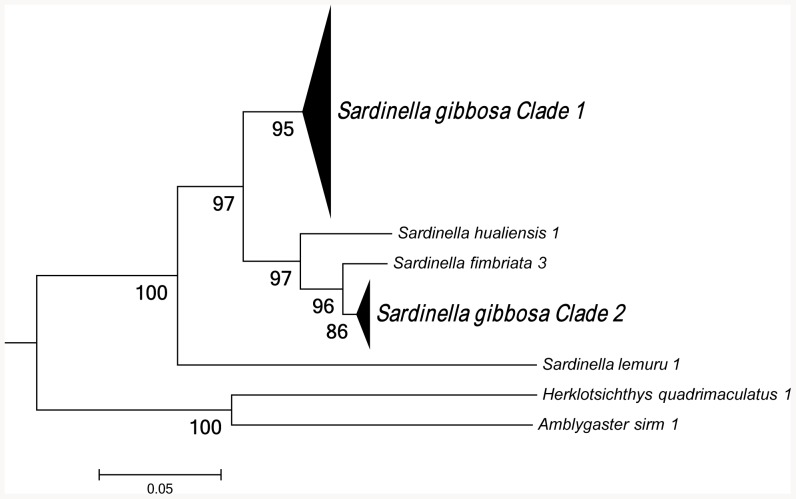
Maximum-likelihood (ML) tree generate from the 1^st^ intron of ribosomal S7 sequences. Bootstrap support values were calculated using 1,000 replicates.

**Figure 4:**
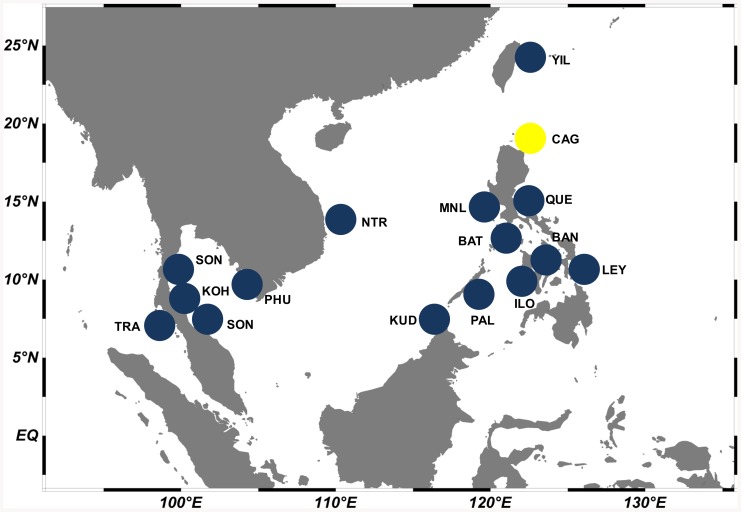
Sampling sites and clade distribution for *S. gibbosa*. Each color represents the cryptic clades. Clade 1 is represented in blue, clade 2 is shaded in yellow.

**Figure 5:**
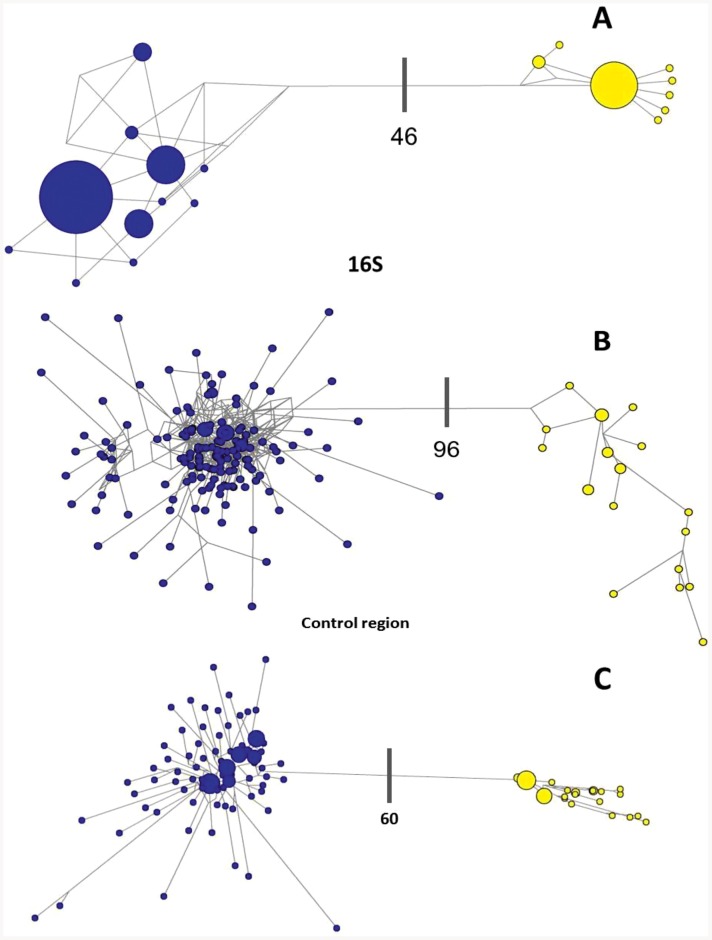
Median-joining network inferred for 16S (A) and control region (B) and S7. Each pie represents a haplotype proportional to its frequency. Thick bars represents step changes between Clade 1 (blue) and Clade 2 (yellow) which is labeled according to the number of base-pair difference.

Maximum-likelihood analysis of 16S rRNA sequences support the existence of two species within *S. gibbosa* (Figure 1). In concordance with 16S data, mitochondrial control region sequences revealed similar clustering (Figure 2). Clustering for both markers exhibited monophyletic clades with high bootstrap support. In addition, nuclear DNA sequences of the first intron of S7 gene revealed a deep divergence between Clade 1 and Clade 2 (Figure 3). None of the phylogenetic analyses indicated that the two morphotypes initially identified as *S. gibbosa* are sister species. Consistent across examined gene regions, genetic distances calculated for both mitochondrial and nuclear gene regions exhibited divergence comparable to species-level differentiation (Table 2). Clade 1 is broadly distributed across the collection sites except at the northernmost locations (Figure 4). In contrast, Clade 2 is geographically restricted to this one northernmost site in Cagayan Province. Further, the single sample from Yilan County, Taiwan did not cluster with Clade 2, despite the site's close proximity with Cagayan Province. However, the current dataset for Taiwan, Vietnam, Thailand and Malaysia are only limited to the mitochondrial 16S gene region. Nevertheless, median joining network for all three markers, at least for Philippine sites, revealed numerous base-pair mutations between Clades 1 and 2 (Figure 5).

**Table 2:** Pairwise genetic distances calculated for each taxa.

16S							
Species	1	2	3	4	5	6	7
*Sardinella gibbosa Clade 1*		0.03	0.05	0.04	0.03	0.08	0.07
*Sardinella gibbosa Clade 2*	0.13		0.02	0.03	0.04	0.09	0.10
*Sardinella fimbriata*	0.22	0.08		0.04	0.07	0.13	0.11
*Sardinella hualiensis*	0.15	0.11	0.16		0.04	0.14	0.14
*Sardinella lemuru*	0.11	0.18	0.27	0.16		0.09	0.06
*Amblygaster sirm*	0.33	0.36	0.50	0.50	0.37		0.12
*Herklotsichthys quadrimaculatus*	0.30	0.37	0.39	0.47	0.25	0.47	

Control region					
Species	1	2	3	4	5
*Sardinella gibbosa Clade 1*		0.07	0.08	0.07	0.06
*Sardinella gibbosa Clade 2*	0.40		0.09	0.12	0.10
*Sardinella fimbriata*	0.52	0.52		0.20	0.12
*Sardinella hualiensis*	0.43	0.64	1.00		0.10
*Sardinella lemuru*	0.40	0.61	0.75	0.66	

S7 intron							
Species	1	2	3	4	5	6	7
*Sardinella gibbosa Clade 1*		0.02	0.03	0.10	0.03	0.13	0.24
*Sardinella gibbosa Clade 2*	0.10		0.01	0.09	0.02	0.15	0.26
*Sardinella fimbriata*	0.10	0.03		0.11	0.02	0.17	0.27
*Sardinella hualiensis*	0.11	0.07	0.08		0.09	0.27	0.53
*Sardinella lemuru*	0.34	0.35	0.38	0.37		0.16	0.48
*Amblygaster sirm*	0.51	0.57	0.63	0.80	0.60		0.09
*Herklotsichthys quadrimaculatus*	0.62	0.72	0.77	1.00	0.95	0.41	

The standard error of mean is shown in the upper right diagonals.

**Figure 6:**
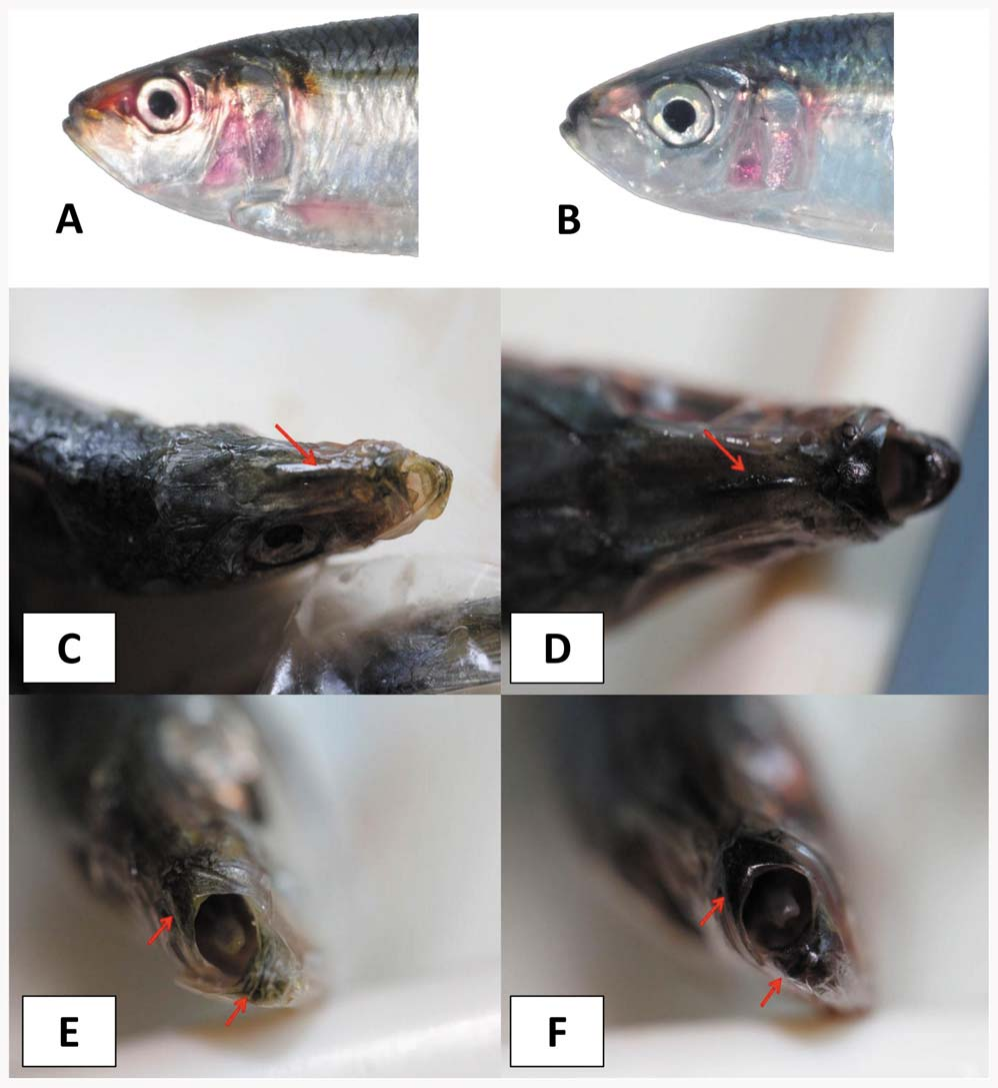
Photos of *Sardinella gibbosa* representing Clade 1 (A) and Clade 2 (B). Between-clade color differences in median frontal line of the head (C–D) and pigmentation of the upper and lower jaw (E–F).

**Figure 7:**
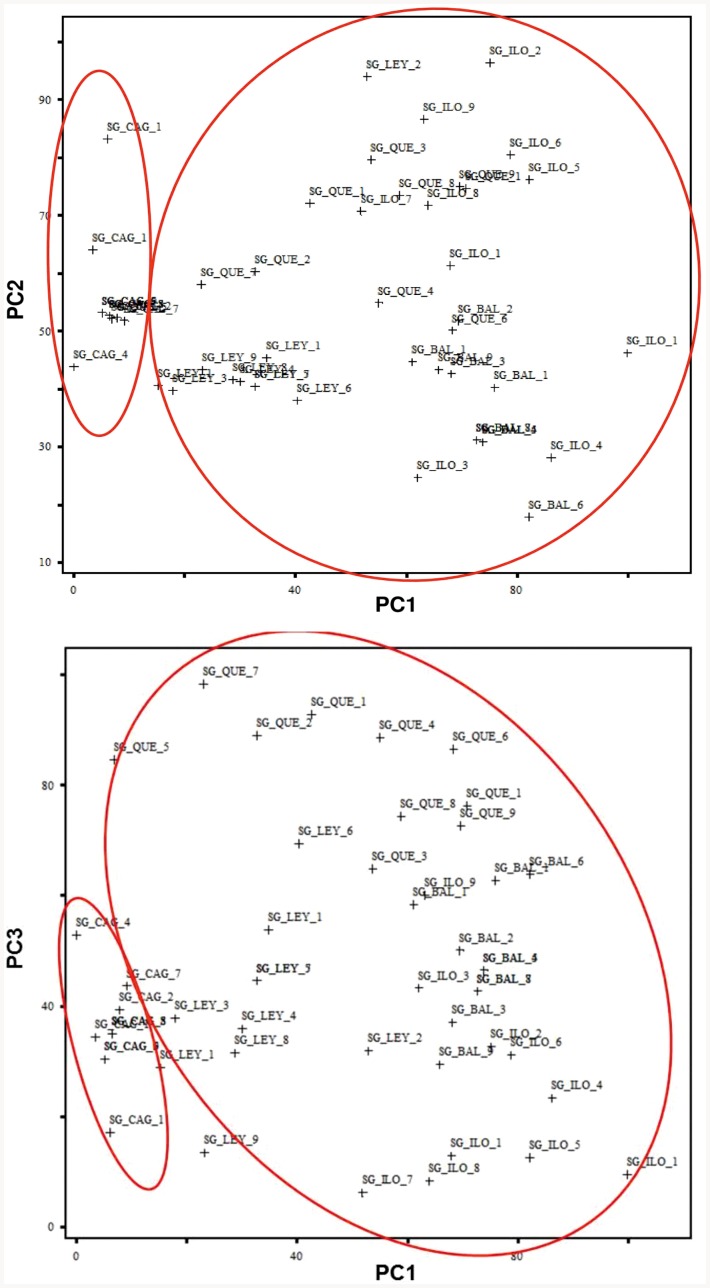
Plots of the first versus the second (A) and the first versus the third (B) principal components (PC) of 60 nominal specimens (n = 10 per population).

All specimens exhibited the diagnostic characters for *S. gibbosa*, including the dark spot at dorsal fin origin. However, head shape and pigmentation of both lower and upper jaws differ between the two clades identified using genetic markers (Figure 6). Principal component analysis (PCA) revealed strong morphometric differentiation in head dimensions (Figure 7). The first four principal components (PC) account for 95.42% of overall variance (PC1 – 48.74%; PC2 – 21.27%; PC3 – 14.94%; PC4 - 10.79%) (Table 3). PC1 was highly correlated with variance in upper jaw length, eye diameter, and post-orbital length, respectively. On the other hand, PC's 2 through 4 were associated with differences in the ratios of head length, upper jaw, and eye diameter. In concordance with the genetic clades, a surprisingly similar clustering was observed in plots of the principal components (Figure 7). Scatter plots for PC1 and PC3 separated collections from Quezon Province into a distinct cluster. Such grouping might indicate a unique sub-population or subspecies in Clade 1. A shorter snout and upper jaw with respect to head length in individuals from the four clades accounted for such clustering in principal component analysis.

**Table 3:** Summary of principal component analysis (eigenvalues and eigenvectors) calculated from 5 morphometric ratios describing head shape.

	PC 1	PC 2	PC 3	PC 4	PC 5
Eigenvalue	2.440	1.063	0.747	0.540	0.213
% variance	48.74	21.27	14.94	10.79	4.27
Eigenvector					
head	0.403	**−0.126**	**0.875**	0.101	**−**0.213
snout	**−**0.130	**0.893**	0.228	**−0.366**	**−**0.011
eye	**−0.515**	0.123	**0.291**	**0.651**	**0.459**
postorbital	**−0.453**	**−0.408**	**0.306**	**−0.652**	**0.331**
upper jaw	**−0.591**	**−**0.077	0.058	0.082	**−0.796**

Loadings with absolute values >0.3 are shown in bold.

Multivariate analysis of morphometric ratios using ANOSIM showed significant variations between head shape of the three genotypic clades (R = 0.486; p<0.01). Differences in snout, post-orbital, and head lengths distinguished the two clades of *S. gibbosa* (Table 4). Strong differentiation in head and upper jaw length accounted for 52.26% of variation between Clade 1 and Clade 2. On the other hand, variations in eye, snout, and post-orbital length contributed 47.74% of overall difference between the two clades. Clade 1 had shorter heads with respect to standard length. Lastly, Clade 2 individuals had the lowest post-orbital length, and subsequently a shorter operculum.

**Table 4:** Summary of 5 morphometric measurements representing variations in head shape.

		Morphometric ratio (% *Ls*)		Clade 1 vs. Clade 2 (% difference)	
Morphometric measurement		Clade 1	Clade 2	Contribution	Cumulative
head length		22.33	24.27	32.00	32.00
upper jaw		40.48	34.62	20.25	52.26
eye		26.82	23.08	18.78	71.04
snout		30.31	30.06	15.74	86.78
post-orbital		40.58	38.46	13.22	100.00

Morphometric ratio refers to each character value as a percentage of standard length (% *Ls*). Contribution and cumulative differences calculated using SIMPER describes, in percentage, each morphometric character's contribution to head shape variation between Clade 1 and Clade 2.

In addition to morphometric difference, Clade 2 has a distinct black pigmentation on the edge of mouth and frontal line between the nostrils (Figure 6). Similar blackish coloration has been observed in the caudal fins of Clade 2. Further, a leaner body characterized Clade 2 in contrast with the more rounded shape of Clade 1 (data not shown). Lastly, Clade 2 lacked the gold stripe across the lateral body wall which characterized Clade 1 (Figure 6).

## Discussion

Many discoveries of cryptic species have been based on prior observations of subtle behavioral, biological, or morphological intra-species variations [5]. However, phenotypic differentiation does not necessarily complement genotypic divergence [9], as evident in the lack of congruence between genetics and diagnostic morphological characters [6], [10], [52]. In extreme cases, morphological variations are randomly shared among genetically distinct lineages within a cryptic species complex [11], [53]. To avoid the inconsistency between genetics and morphology, the straightforward approach is to identify cryptic species using multi-locus genetic data [54], [55], a method that can help avoid the pitfalls of morphological species delimitation [12]. Cryptic species identified through genetic clustering can then be bolstered by support from additional morphological or biological traits.

In *S. gibbosa*, molecular evidence from both mitochondrial and nuclear DNA strongly supports two cryptic species. Clade 1 is widely distributed throughout the Philippine islands, while Clade 2 is geographically restricted to the Cagayan Province. Such allopatric distribution has been reported to occur in other cryptic species [15], [16]. In addition, the two clades exhibited the same clustering for all markers (Figures 1–3), a finding consistent with the ‘genotypic-clustering’ species definition [12], and substantiated by agreement between multi-locus genotypic data [56], [57]. Likewise, a lack of reciprocal monophyly in Clades 1 and 2 showed that the two lineages are different and not sister species. Clades 1 and 2 were paraphyletic with each other, a phylogenetic pattern that commonly occurs among cryptic species [58], [59]. In addition, the 10–40% genetic distances between cryptic clades are comparable to species-level differences (Table 2) [60]. In some pairs, genetic distances for 16S rRNA and S7 intron of *S. gibbosa* exceed levels typically distinguishing closely-related species [60]. It is also interesting to note that Clades 1 and 2 do not have shared haplotypes in both the conserved 16S rRNA and the polymorphic mitochondrial control region (Figure 5). Consistent patterns in both maternally and bi-parentally inherited genetic markers demonstrate a lack of gene flow between the two cryptic species. Such patterns fall within the framework of the general species concept for two unique species [1]. Hence, genetic information from both mitochondrial and nuclear DNA presents solid evidence for two biologically distinct species.

Distinct morphometric variations in head shape characterized both Clade 1 and 2 of the *S. gibbosa* species. Multivariate analysis of head measurements revealed clustering comparable to the genetic clades. Similar clustering due to head shape variations have characterized sub-species within a sardine [23], [61], a result later confirmed by molecular evidence from mitochondrial data [62]. Morphological differences between closely related sardines are often characterized by slight differences in measurements or meristic counts, resulting in an ambiguous and often controversial taxonomic status [23]. For instance, the sister sardine species *Sardinella tawilis* and *Sardinella hualiensis* share diagnostic characters; and excluding habitat preference, differ only in head length and lower gillraker count [63]. However, intra-species morphological variations in sardines are often presumed to be an artifact of localized adaptations to environment, due to a lack of support from significant genetic differentiation between morphological forms [64], [65]. In contrast, the morphological disparity between the cryptic clades of *S. gibbosa* complements genetic divergence (Figure 6), and thus is not a mere localized ecological adaptation. Lastly, clustering in PCA due to head shape in Clades 1 and 2 of *S. gibbosa* falls within the phenetic-clustering species delimitation, as there is a lack of intermediate forms in between the two clades [12], [66].

Combined, genetic and morphological data reveal a hidden diversity in a common and commercially important sardine. Our findings expand the previous investigations on the biology, ecology, and morphology of *S. gibbosa* that alluded to a cryptic diversity [25], [31]. Discovery of new fish species in the Northern Philippines [67], [68] including a sardine beyond its previously known distribution [69], suggests that this region harbors undocumented and unique fauna. Such a pattern presents the possibility that Clade 2 might be a new species. Alternatively, Clades 1 and 2 might be previously documented synonyms of *S. gibbosa* [23]. Based on geographic proximity and morphological similarity, the most likely candidate synonym is *S. taiwanensis* [35]; however, further scrutiny of type specimens is necessary for validation. Nevertheless, the findings in this study demonstrate that a combination of both morphological and genetic data is essential to assess diversity in taxonomically ambiguous sardines. Here, strong evidence of two ecologically similar, but genetically and morphologically distinct species warrants appropriate management strategies for separate sardine fisheries.
